# Diversity and Community Composition of Methanogenic Archaea in the Rumen of Scottish Upland Sheep Assessed by Different Methods

**DOI:** 10.1371/journal.pone.0106491

**Published:** 2014-09-24

**Authors:** Timothy J. Snelling, Buğra Genç, Nest McKain, Mick Watson, Sinéad M. Waters, Christopher J. Creevey, R. John Wallace

**Affiliations:** 1 Rowett Institute of Nutrition and Health, University of Aberdeen, Bucksburn, Aberdeen, United Kingdom; 2 Department of Animal Nutrition and Nutritional Diseases, Faculty of Veterinary Medicine, Ondokuz Mayis University, Samsun, Turkey; 3 ARK Genomics, The Roslin Institute, Easter Bush, Midlothian, United Kingdom; 4 Animal and Bioscience Research Department, Animal and Grassland Research and Innovation Centre, Teagasc, Grange, Dunsany, Co. Meath, Ireland; 5 Institute of Biological, Environmental and Rural Sciences, Aberystwyth University, Aberystwyth, Ceredigion, United Kingdom; University of Illinois, United States of America

## Abstract

Ruminal archaeomes of two mature sheep grazing in the Scottish uplands were analysed by different sequencing and analysis methods in order to compare the apparent archaeal communities. All methods revealed that the majority of methanogens belonged to the Methanobacteriales order containing the *Methanobrevibacter, Methanosphaera and Methanobacteria* genera. Sanger sequenced 1.3 kb 16S rRNA gene amplicons identified the main species of *Methanobrevibacter* present to be a SGMT Clade member *Mbb. millerae* (≥91% of OTUs); *Methanosphaera* comprised the remainder of the OTUs. The primers did not amplify ruminal Thermoplasmatales-related 16S rRNA genes. Illumina sequenced V6–V8 16S rRNA gene amplicons identified similar *Methanobrevibacter* spp. and *Methanosphaera* clades and also identified the Thermoplasmatales-related order as 13% of total archaea. Unusually, both methods concluded that *Mbb. ruminantium* and relatives from the same clade (RO) were almost absent. Sequences mapping to rumen 16S rRNA and *mcrA* gene references were extracted from Illumina metagenome data. Mapping of the metagenome data to16S rRNA gene references produced taxonomic identification to Order level including 2–3% Thermoplasmatales, but was unable to discriminate to species level. Mapping of the metagenome data to *mcrA* gene references resolved 69% to unclassified Methanobacteriales. Only 30% of sequences were assigned to species level clades: of the sequences assigned to *Methanobrevibacter*, most mapped to SGMT (16%) and RO (10%) clades. The Sanger 16S amplicon and Illumina metagenome *mcrA* analyses showed similar species richness (Chao1 Index 19–35), while Illumina metagenome and amplicon 16S rRNA analysis gave lower richness estimates (10–18). The values of the Shannon Index were low in all methods, indicating low richness and uneven species distribution. Thus, although much information may be extracted from the other methods, Illumina amplicon sequencing of the V6–V8 16S rRNA gene would be the method of choice for studying rumen archaeal communities.

## Introduction

Methanogenic archaea are part of the anaerobic microbial community of the rumen. Though less abundant than the ruminal bacteria they have received a great deal of attention due to their ability to synthesise methane. Methanogenesis from the rumen can occur via three known metabolic pathways although the hydrogenotrophic reduction of CO_2_ by H_2_ predominates [1]. The production of methane by ruminants represents a loss of energy to the animal [2], and with 3.6 billion domestic ruminants and 1.1 billion sheep globally enteric methane is also believed to be a significant contributor to anthropogenic global greenhouse gas (GHG) emissions [3]. In Scotland, sheep farming represents an important sector of the agricultural industry, with approximately 2.75 million currently registered breeding ewes [4]. Even at a national level, methane production from sheep has been recognised as a significant challenge to meeting proposed targets of lowering GHG emissions by 20% by 2020 [5]. Assessment of the archaeal community is a prerequisite for rational manipulation of the ruminal microbiota to lower methane emissions.

Molecular analyses of the ruminal archaea have been based mainly on 16S rRNA gene amplicons, revealing the methanogen diversity and phylogeny in a number of ruminant species, including cattle [6]–[9], alpaca [10], reindeer [11], [12], domesticated red deer [13], and water buffalo [14],[15]. Archaeal communities in sheep have been assessed previously in New Zealand [13], France [16], Japan [17], Australia [18], [19] and Venezuela [20]. The methyl-coenzyme reductase A (*mcrA*) gene involved in the methanogenesis pathway has provided an alternative marker to identify ruminal archaea in lambs [21] and cattle [22], providing good correlation of the different phylogenetic analyses. In almost all studies, the rumen methanogens have been predominantly assigned to the order Methanobacteriales. These methanogens have been divided into two major and correlated groups of Methanobrevibacter species (*Mbb.*) [23], *Mbb. smithii*, *gottschalkii*, *millerae* and *thaueri*, referred to as the SGMT clade, and *Mbb. ruminantium* and *olleyae,* referred to as the RO clade. With amplicon based methods, there have been concerns highlighting the comparability of the different community analyses due to primer bias [24] and also a failure to appreciate the role of the Rumen Cluster C (RCC) clade, related to the order Thermoplasmatales [25].

Microbial diversity can also be assessed using metagenomic methods where function and taxonomy can be obtained from a single dataset. Moreover, using extracted genomic DNA as a starting material avoids potential amplicon sequencing biases. In studies comparing amplicon and metagenomic methods, the metagenomic analysis has compared well to synthetic reference archaeal and bacterial communities [26].

The aim of the present study was to characterise by different methods the community of methanogenic archaea in the rumen of the economically and environmentally important sheep grazing on Scottish upland pastures. Although the main properties of the community were consistent across different methods, important differences emerged in relative abundance and more detailed taxonomic identification.

## Materials and Methods

### Animals and sampling

All the animal experimentation for this study was carried out under the conditions set out by a UK Home Office licence no. 604028, procedure reference number 8. Samples of digesta were taken from the rumens of two mature Finn-Dorset cross sheep (Sheep A and Sheep B), each fitted with a ruminal cannula. The animals were grazing a mixed pasture at Glensaugh, Scotland (altitude 300 m, mean annual temperature 7.5°C, rainfall 1130 mm) in June 2011. Approximately 50 ml of digesta were taken from each animal via a 20-mm diameter plastic tube, homogenized to detach the fibre adherent microbes and strained through two layers of gauze. Aliquots of 20 ml of the filtrate were transferred into sterile plastic containers and placed on dry ice for transportation and then stored in a freezer at −20°C.

### Sanger amplicon sequencing of 16S rRNA gene (SA *rrn*)

Separate clone libraries were constructed for each of the two sheep from the respective digesta samples. DNA extraction was carried out using a method based on repeated bead beating using a Mini-Beadbeater (Biospec Products) plus column filtration (RBB+C) [27]. Column filtration was carried out using the reagents and spin filter column provided with the QIAamp DNA Stool Mini Kit according the manufacturer’s instructions (Qiagen, GmbH).

Methanogenic archaeal 16S rRNA genes (*rrn*) were amplified by PCR using the universal primers Arch f364 and Arch r1386 designed by Skillman *et al*. [28]. The amplicons were ligated into TOPO TA pCR 2.1 cloning vector (Life Technologies) and transformed into TOP10 chemically competent *Escherichia coli*. Positive transformed colonies were selected at random and the recombinant plasmids sequenced on a Beckman Coulter CEQ8000 platform following clonal amplification using a QuickStart dye terminator master mix (Beckman Coulter Inc.) with M13 forward and reverse sequencing primers and the universal archaeal primers Met448F, Met448R, Met1027F and Met1027R [29].

Contigs were assembled by initially mapping the sequence fragments against a reference sequence obtained from the Ribosomal Database Project [30] of a type strain of *Methanobrevibacter ruminantium* (Acc. No. AY196666) [29]. The overlapping regions were inspected for mismatches or gaps and corrected to generate consensus sequences with the minimum number of ambiguities. Vector contamination was identified and removed after comparing the sequences to the UniVec database using VecScreen (NCBI).

Sequences were checked for possible chimeras using Bellerophon [31] and a non-redundant set of operational taxonomic units (OTUs) was generated *de novo* from a distance matrix of the sequences constructed using the PHYLIP package dnadist [32] and clustered at values >98% sequence similarity using mothur) at values >98% sequence similarity [33]. Representative sequences for each OTU were entered as queries and searched using BLASTn against the NCBI GenBank nucleotide database [34] to assign taxonomy to the nearest valid species.

### Illumina amplicon sequencing of 16S rRNA gene (IA *rrn*)

16S rRNA gene amplicons were generated using DNA Free Sensitive Taq polymerase (Bioron, GMbH) and primers Ar915aF and Ar1386R by Kittelmann *et al*., (2013) [23]. Cycling conditions were: 94°C (2 min), then 30 cycles of 94°C (10 s), 68°C (20 s), 72°C (1 min). Amplicons were purified with a Qiaquick PCR purification kit (Qiagen, GmbH) according to manufacturer’s instructions. 500 ng of each purified amplicon was then end repaired using the NEBNext End Repair Module (New England Biolabs Inc.). End repaired amplicons were purified with a Qiaquick PCR purification kit (Qiagen, GmbH) and a single adenine was added to the 3′ ends using the NEBNext dA-Tailing Module (New England Biolabs Inc.). Partial Truseq standard paired end Illumina adapters with 6 bp barcodes (Integrated DNA Technologies) were ligated to the adenylated amplicons using the Quick Ligation Kit (New England Biolabs Inc.) and resulting adapter-ligated amplicons were purified with a Qiaquick PCR purification kit (Qiagen, GmbH). Full length adapter-ligated libraries were then generated by 7 cycles of PCR using Truseq paired end PCR primers (Integrated DNA Technologies) and Kapa HiFi Hotstart ready mix (Kapa Biosystems Inc.), then purified with Qiaquick PCR purification kit (Qiagen, GmbH). Cycling conditions were 95°C (2 min), then 7 cycles of 98°C (20 s), 58°C (15 s), 72°C (20 s) and a final extension step of 72°C (5 min). The resulting full-length libraries were denatured and diluted to 6 pM and spiked with 30% v/v denatured 12 pM PhiX control library (Illumina Inc.). The spiked 16S rRNA gene amplicon library was run on an Illumina MiSeq with 500 cycle Miseq Reagent Kit v2 sequencing chemistry (Illumina Inc.). Using the proprietary Illumina software suite, the reads were de-multiplexed and filtered to retain only those containing both 5′ and 3′ primer sequences. Paired-end reads were joined using the “fastq-join” command from ea-utils (http://code.google.com/p/ea-utils), specifying no difference between the two reads in the overlapping region (-p 0) and a minimum overlap of 10 nt (-m 10).

Clustering and determination of Operational Taxonomic Units (OTUs) were performed using CD-HIT-OTU [35] with a 97% identity cut-off. Taxonomic classification was carried out by submitting the representative sequence from each OTU to the RDP classifier [30] and individually queried using BLASTn against GenBank to assign taxonomy to the nearest valid species.

### Illumina metagenome sequencing (IM *rrn*, IM *mcrA*)

Metagenomic sequences were generated from genomic DNA prepared in the form of 101-nucleotide (nt) paired-end reads using a HiSeq 2000 instrument (Illumina Inc.) at ARK-Genomics (University of Edinburgh). DNA from each sample was sheared randomly using an ultrasonicator (Covaris Inc.) and libraries for sequencing were constructed using a TruSeq DNA sample preparation kit (Illumina Inc.). Illumina metagenome 16S rRNA gene sequence dataset (IM *rrn*) was produced as follows: a rumen 16S rRNA gene database was constructed from Bacteria and Archaea references downloaded from the Ribosomal Database Project (RDP) website [30]. Sequences were selected from ≥1200 bp in length and with the quality tag ‘Good’ and with the keyword ‘rumen’. Illumina metagenome sequences were clustered and aligned to this database using Novoalign (www.novocraft.com) using the ‘-r All’ parameter setting to report all alignments. Where a fragment pair aligned to a single reference, the full taxon was reported. Where a fragment pair aligned equally well to multiple references, the lowest common taxon was reported.

The Illumina metagenome *mcrA* gene sequence dataset (IM *mcrA*) was prepared with a similar database, containing a complete set of *mcrA* genes, downloaded from the FunGene repository (http://fungene.cme.msu.edu/hmm_details.spr?hmm_id=16). OTU picking and taxonomic identification followed the same analysis method used previously for the IM *rrn* dataset.

### Community diversity analysis

Good’s depth of coverage (C) was assessed using the formula C = 1−(n/N) where n is the number of singletons and N the total number of clones sequenced [36]. The diversity of the OTUs for each sheep was calculated using the Shannon index (H′) as a summary measurement of species richness and evenness [37] and Chao1 indices as an estimate of the likely number of species [38]. Multiple rarefaction curves were also generated to assess depth of sequencing and species richness.

### Phylogenetic analysis

Full multiple alignment and pairwise comparison of the OTU representative sequences was carried out using ClustalW [39]. A phylogenetic tree was constructed with MEGA5 [40] using the Neighbor-Joining method [41] with Jukes Cantor nucleotide substitution model and bootstrap resampling 1000 times.

## Results

### Sequencing and taxonomic identification

**Figure 1:**
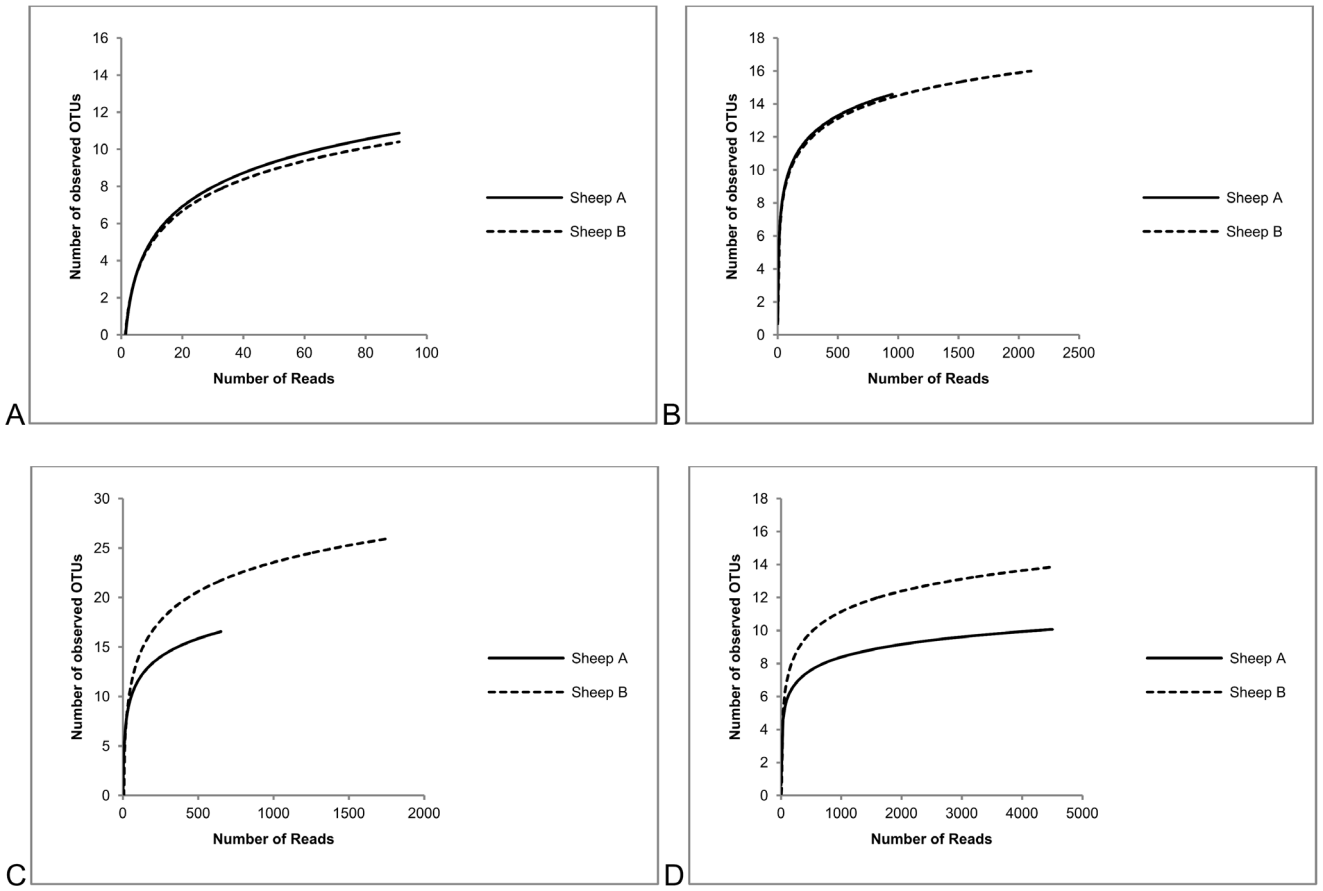
Multiple rarefaction collectors curves. Observed number of OTUs for different sequencing and analysis methods: A. SA *rrn*. B. IM *rrn*. C. IM *mcrA*. D. IA *rrn.*

The Illumina HiSeq sequencing effort generated 307 million reads for Sheep A and Sheep B with a total of 216,038 reads mapped to prokaryote 16S rRNA references. Illumina metagenome *mcrA* gene (IM *mcrA*) and Illumina metagenome 16S rRNA gene (IM *rrn*) sequencing methods mapped 2442 reads and 3107 reads to methanogenic archaeal references and assigned them to 31 OTUs and 18 OTUs respectively. The assembly of the Sanger amplicon sequenced 16S rRNA amplicon (SA *rrn*) sequences produced 203 contigs of sequence length >1000 nt after vector removal, and clustering produced a set of 21 OTUs (RINH01-RINH21; Table 1). Representative sequences were submitted to EMBL, returning accession numbers HE858590 to HE858610. The Illumina amplicon sequencing of 16S rRNA genes (IA *rrn*) method produced 10982 reads with average sequence length 483 nt and clustered to 16 OTUs. The representative sequences of OTUs containing more than five reads per OTU were retained for phylogenetic analysis (T01–T10; Table 1). Alpha diversity statistics for each method including multiple rarefaction curves, Shannon index, Chao1 estimated number of species and Good’s depth of coverage (C) are presented in Figure 1 and Table 2.

**Table 1:** Methanogenic archaea of Sheep A and Sheep B.

16S rRNA OTU	Length (nt)	Reads Sheep Ag2	Reads Sheep Bg2	Nearest valid species	Acc No.	% sequence identity
**Sanger**						
RINH01	1030	84	63	*Methanobrevibacter millerae*	NR042785	98
RINH02	1031	1	0	*Methanobrevibacter millerae*	NR042785	99
RINH03	1029	4	14	*Methanobrevibacter millerae*	NR042785	99
RINH04	1027	3	1	*Methanosphaera stadtmanae*	CP000102	96
RINH05	1027	3	2	*Methanosphaera stadtmanae*	CP000102	97
RINH06	1083	2	8	*Methanobrevibacter millerae*	NR042785	99
RINH07	1030	2	1	*Methanobrevibacter smithii*	CP000678	95
RINH08	1027	2	0	*Methanosphaera stadtmanae*	CP000102	97
RINH09	1028	0	1	*Methanosphaera stadtmanae*	CP000102	96
RINH10	1036	0	1	*Methanobrevibacter millerae*	NR042785	99
RINH11	1028	0	1	*Methanosphaera stadtmanae*	CP000102	97
RINH12	1104	0	1	*Methanosphaera stadtmanae*	CP000102	94
RINH13	1032	0	1	*Methanobrevibacter millerae*	NR042785	98
RINH14	1034	0	1	*Methanobrevibacter millerae*	NR042785	97
RINH15	1027	1	0	*Methanosphaera stadtmanae*	CP000102	96
RINH16	1038	1	0	*Methanobrevibacter millerae*	NR042785	97
RINH17	1125	1	0	*Methanobrevibacter millerae*	NR042785	94
RINH18	1027	1	0	*Methanosphaera stadtmanae*	CP000102	95
RINH19	1029	1	0	*Methanosphaera stadtmanae*	CP000102	94
RINH20	1037	1	0	*Methanobrevibacter millerae*	NR042785	98
RINH21	1055	0	1	*Methanobrevibacter millerae*	NR042785	97
**Illumina**						
T01	492	3655	4630	*Methanobrevibacter millerae*	NR042785	99
T02	488	559	664	*Aciduliprofundum boonei*	NR074217	84
T03	488	345	545	*Methanosphaera stadtmanae*	CP000102	97
T04	486	153	30	*Aciduliprofundum boonei*	NR074217	82
T05	490	10	147	*Methanobrevibacter ruminantium*	NR074117	98
T06	486	90	40	*Methanosphaera stadtmanae*	CP000102	96
T07	486	27	28	*Methanosphaera stadtmanae*	CP000102	95
T08	488	0	27	*Picrophilus torridus*	NR074187	83
T09	478	5	9	*Methanosarcina barkeri*	JQ346756	100
T10	477	0	6	*Methanoculleus palmolei*	NR028253	99

Sanger and Illumina 16S rRNA amplicon OTUs clustered at 97% sequence identity. Taxonomic classification to the nearest valid species by BLASTn search of representative sequences to the GenBank nucleotide database.

**Table 2:** Comparison of methanogenic archaeal diversity and depth of coverage for Sheep A and Sheep B.

	Sanger 16S		Illumina Metagenome 16S		Illumina Metagenome mcrA		Illumina 16S Amplicon	
	Sheep A	Sheep B	Sheep A	Sheep B	Sheep A	Sheep B	Sheep A	Sheep B
Shannon (H′)	1.5	1.8	2.5	2.4	1.9	2.1	1.28	1.29
Chao1	19	31	18	17	20	35	10	18
Good (C)	0.67	0.57	0.83	0.88	0.92	1.0	1.0	0.75

Shannon index (H′), Chao1 estimated number of species and Good’s statistic (C).

**Figure 2:**
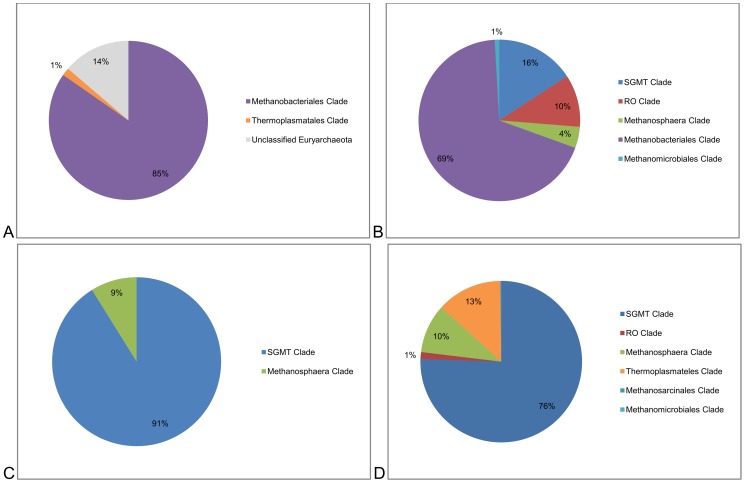
Relative distribution of methanogenic archaeal clades for different sequencing and analysis methods: A. SA *rrn*. B. IM *rrn*. C. IM *mcrA*. D. IA *rrn.*

Taxonomic classification in all sequencing and analysis methods assigned all OTUs to the methanogenic archaea phylum Euryarchaeota. The taxonomic summaries for each method are presented in Table 1 and Figure 2. The majority of OTUs (85%–100%) obtained in all methods were assigned to the order Methanobacteriales. In detail, the resolution and relative abundance of the various clades varied between methods. However, a dominance of members of the genus *Methanobrevibacter*, and in particular the SGMT clade including the species *Mbb. smithii*, *Mbb. gottschalkii*, *Mbb. millerae* and *Mbb. thauerii*, was apparent (Figure 2). Notable differences were seen in the relative abundance of members of the RO clade (*Mbb. ruminantium* and *Mbb. olleyae*), with 10% assigned by the IM *mcrA* method, 1% by the IA *rrn* method and none from the SA *rrn* method. Taxonomic identification to species level was not possible by mapping the Illumina Metagenome to 16S rRNA gene references (IM *rrn* method). Therefore, the proportion of SGMT and RO clades within the Methanobacteriales could not be determined with this method. The proportion of the Thermoplasmatales also varied between methods, from none detected using SA rrn and IM *mcrA* methods to 1% with the IM *rrn* method and 13% with the IA *rrn* method. The *Methanosphaera* clade was somewhat less variable between methods, where identified, with relative abundance of 4% detected with IM *mcrA*, 9% SA *rrn* and 10% IA *rrn* respectively.

### Phylogenetic analysis

**Figure 3:**
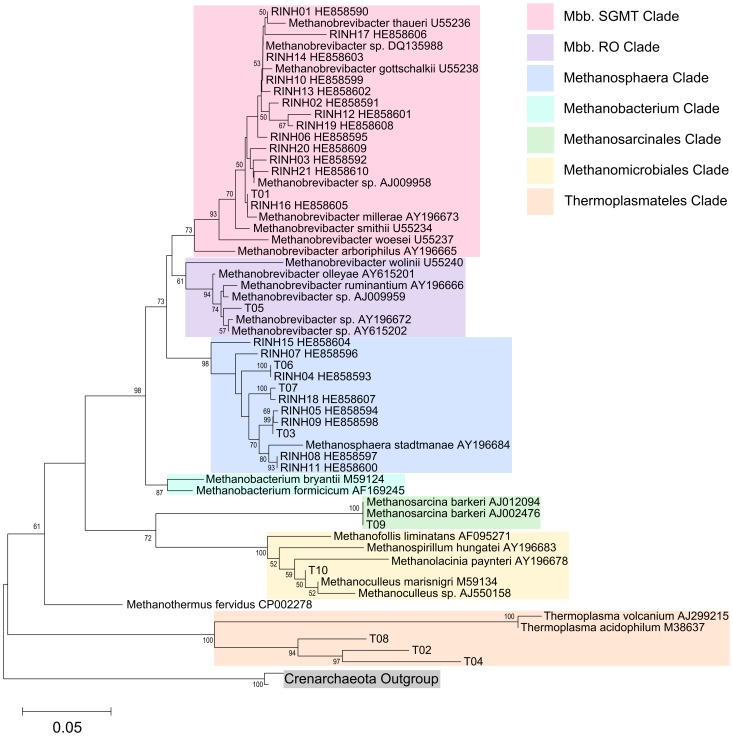
Phylogenetic analysis of SA *rrn* OTUs (RINH01–RINH21) and IA *rrn* OTUs (T01–T10). Placement of representative sequences of the present study in clades indicated with additional reference sequences obtained from GenBank. A sequence related to the Crenarchaeota phylum (Acc. No. AF418935) was used as an outgroup. Full multiple alignment using ClustalW and a consensus tree was constructed using the Neighbor-Joining method with the Jukes-Cantor substitution model. The trees were bootstrap resampled 1000 times with branch values ≥50% shown. Scale shows 0.05 nucleotide substitutions per nucleotide position.

Phylogenetic analysis was carried out on the data where representative sequences were available and included data from the SA *rrn* and IA *rrn* methods. Full pairwise alignment of both the IM rrn and IM mcrA datasets was confounded by the fragmented nature of the reads. Sequences were mapped to random positions on the 16S rRNA gene and overlapping regions were variable or absent. Taxonomic identification was confirmed by placement in a branch containing valid species from the respective clades (Figure 3).

Phylogenetic analysis placed the most abundant SA *rrn* OTU (RINH01) in a group containing the methanogenic archaeal species *Mbb. thaueri* (U55236). This and a further 12 OTUs containing 92% of the sequences were all placed within the monophyletic SGMT clade [10], [15]. The remaining nine OTUs containing 8% sequences were assigned to a clade containing the single valid species *Methanosphaera stadtmanae* (AY196684) (Figure 3). Similarly, the most abundant IA *rrn* OTU (T01) was placed near *Mbb. millerae*. This was the only IA *rrn* OTU placed within the SGMT clade although it accounted for over 75% of the relative abundance of methanogens detected using this method. The apparent richness was also greater with 1% OTU representatives assigned to the *Mbb.* RO clade and the Thermoplasmatales, Methanosarcinales and Methanobacteriales orders.

## Discussion

Characterisation and measurements of diversity of methanogenic archaea were made from the ruminal digesta of two sheep kept on Scottish upland grazing. Different sequencing and analysis methodologies were applied to assess sequencing coverage, detection of the different taxa and effectiveness for calculating species diversity in each case. The methods used here were divided into two broad categories: an untargeted approach, where species are inferred by mining metagenomic data and mapping onto a reference database, and a targeted approach using PCR to amplify of a marker gene, alignment and clustering at specified sequences identity. The metagenome is the total gene content of an environmental microbiota at a given point in time [42]. Therefore, mapping the sequences to a specially curated reference gene database can provide a direct and representative measurement of the microbiota. The limitations are that metagenomic data can contain sequence fragments from any region of the target gene. This means that the information contained may not extend to cover the important hypervariable regions needed to make detailed taxonomic assignation.

Measuring the diversity of an environmental microbiota using amplicon sequencing whether based on a Sanger or NGS platform has also been subject to criticism. With this approach, results can be influenced by PCR amplification bias [43], [44], choice of primers [24] and data analysis method [45]. However, it remains an established approach for measuring microbial communities with abundant resources of target gene databases [30], [46], [47] and analysis software [33], [48].

To the best of our knowledge, methanogenic archaea of the phylum Crenarchaeota have been found in the rumen in one reported instance [49]. Otherwise the taxonomic richness of the ruminal archaeome is relatively poor compared to the bacteria [50] and can be summarised by a single phylum, the Euryarchaeota and four orders: Methanomicrobiales, Methanosarcinales, Methanobacteriales and the Thermoplasmatales (RCC). The latter, recently renamed Methanoplasmatales [51], is implicated in methane emissions in the rumen, possibly from methylamines [25]. Typically, the Methanobacteriales have been shown to be dominant in the rumen of sheep or lambs in a number of studies with members of the SGMT, RO, *Methanosphaera* spp. and *Methanobacterium* spp. clades found in varying relative abundance [20], [21]. The exception to this has been methanogens from the rumen of sheep from Queensland, Australia where the Thermoplasmatales were the major order of methanogens [19].

The results presented here showed a clear majority of the Methanobacteriales using all methods. Mapping of the Illumina metagenome sequences to the rumen 16S rRNA database (IM *rrn*) identified 85% to this order with a further 1% was also mapped to the Thermoplasmatales. This method was severely limited in identifying OTUs with no detailed genus or species clades identified. The lack of resolution would be due in part to the absence of hypervariable regions offering the necessary taxonomic resolution in the sequence fragments.

This issue was also apparent after mapping the Illumina metagenome sequences to the *mcrA* reference database (IM *mcrA*). Here, 69% of the reads were assigned to Methanobacteriales. This method also detected a small proportion of Methanomicrobiales and was also able to identify some genera and species level clades. The failure to detect Thermoplasmatales may have been a result of the limited size and scope of the *mcrA* gene reference database [52].

The Sanger 16S rRNA amplicon sequencing methodology (SA *rrn*) benefited from high quality sequences, long read length and high resolution for taxonomic classification. OTUs for the entire archaeome were mapped to species level clades with members of the *Mbb*. SGMT clade predominating, with the highest number of reads related to *Mbb. millerae* and the remainder members of the *Methanosphaera* clade. SA *rrn* did not detect OTUs from the Thermoplasmatales related order. With any amplicon based method, detection of all the representative members of a microbial community depends on genuinely universal primers. Even a single base mismatch, particularly at the 3′ region can seriously affect primer annealing and bias the measurement of the microbial community [24]. This method in general is also limited by the number of sequences that can be produced in a single sequencing run and the calculation of Good’s coverage (C) of 57%–67% indicated the need for increased sequencing effort. While falling well short of the upper limit of what can be achieved using this method, the 203 near full length SA *rrn* sequences presented here represented a reasonable sequencing effort for a single study. Characterisation of the methanogens in the gut of pigs and humans have yielded 763 and 1524 sequences respectively [53], [54], the latter being a subset of 13355 prokaryotic *rrn* sequences. At this scale, the demands on time and cost begin to have an influence on practicality of Sanger sequencing compared to next generation sequencing methods.

The shorter average read length (483 nt) of the IA *rrn* method did not seem to affect the identification to genus and species level clades with SGMT dominant and the highest number of reads related to *Mbb. millerae*. *Mbb. ruminantium*, a member of the RO clade that was missed by the Sanger method was also detected albeit in small proportion (1%). In comparable studies, the proportions of these major methanogen clades between animals vary inversely [23]. However, the extreme bias toward SGMT was unusual to the Scottish sheep sampled here. The Thermoplasmatales clade was detected at 14% the highest proportion of all methods and the high coverage extended to detecting a few reads assigned to the Methanosarcinales and Methanomicrobiales clades, albeit making up less than 1% of the total archaeome.

An effective molecular method used to characterise the rumen methanogen community must have sufficient resolution to separate taxa at the minimum of genus and with sufficient depth to determine the presence and relative abundance of rare taxa in a population that is unevenly distributed and dominated by a few species. With appropriate primers, a high throughput amplicon sequencing strategy is currently the best way to assess the rumen methanogen community and in this study Illumina paired-end amplicon sequencing (IA rrn) effectively represented the subtle diversity of the ruminal archaeome.

The assessment and validation of the different methods presented here will serve as a guide to selecting the best approach for characterising methanogenic archaea in the rumen. Both microbiome and metagenomic methodologies will be essential tools as part of the investigation of the role of rumen microbiota in methane emissions and global climate change. Methane has been identified as a potent greenhouse gas with 27 times warming potential than CO_2_ [55], and enteric methane emissions derived from the ruminal archaea have been estimated at 20–25%, making it the largest anthropogenic source [56]. Therefore, establishing an accurate and reliable method to characterise the methanogenic archaea in the rumen is an important step in the efforts to help mitigate the environmental impact of global livestock production.
